# Use of Castor Bean Meal, Biodiesel Industry Coproduct, in A Lamb Production System Using Creep-Feeding in Brazil

**DOI:** 10.3390/ani10081250

**Published:** 2020-07-23

**Authors:** Marco A. S. Novaes, Cristina M. Veloso, Otávio H. G. B. D. Siqueira, Matheus F. L. Ferreira, João V. R. Lovatti, Hinayah R. Oliveira, Camila S. Cunha, Tathyane R. S. Gionbelli, Claudio J. B. Espeschit, Henry D. R. Alba, Gleidson G. P. de Carvalho

**Affiliations:** 1 Animal Science Department, Federal University of Viçosa, Viçosa 36570-900, Minas Gerais, Brazil; marcoaurelioschiavo@gmail.com (M.A.S.N.); otavio.hbarbosa@gmail.com (O.H.G.B.D.S.); matheus.fellipe234@gmail.com (M.F.L.F.); lovatti.jvr@gmail.com (J.V.R.L.); hinayah@gmail.com (H.R.O.); tathytt@yahoo.com.br (T.R.S.G.); espeschi@ufv.br (C.J.B.E.); 2 School of Veterinary Medicine and Animal Science, Universidade Federal do Mato Grosso do Sul, Campo Grande 79070-900, Mato Grosso do Sul, Brazil; camila.cunha@ufms.br; 3 Animal Science Department, Federal University of Bahia, Salvador 40170-110, Bahia, Brazil; harrydoo@gmail.com (H.D.R.A.); gleidsongiordano@yahoo.com.br (G.G.P.d.C.)

**Keywords:** alternative feed, animal nutrition, protein, ricin, ruminant

## Abstract

**Simple Summary:**

Economically, feed costs are the most expensive variable in the ruminant industry. Therefore, finding an alternative feed resource to decrease these costs is necessary. Castor bean is an oilseed used in the growing biodiesel industry from which the castor bean meal is obtained; however, the ricin content of the castor bean meal may cause animal poisoning. Thus, it is necessary to detoxify the castor bean meal before using it in animal feed. A creep-feeding method is a supplement to the actual alimentation of young animals and as a complement; its economic value must be lower. This study aimed to evaluate the replacement of soybean meal by detoxified castor bean meal on intake, digestibility, body weight gain, and creep-feeding method effectiveness using castor bean meal in lambs. It was found that replacing soybean meal by detoxified castor bean meal does not compromise the development of animals, while the use of creep feeding positively affects their development. The soybean meal can be replaced by detoxified castor bean meal in a creep-feeding method for lambs.

**Abstract:**

This study aimed to evaluate the replacement of soybean meal by detoxified castor bean meal on the intake and digestibility of nutrients, body weight gain, carcass yield, physiological and urinary parameters, and creep-feeding method effectiveness. For this trial, 43 male lambs were used, Dorper × Santa Inês, with an average initial body weight of 7.95 kg, 15 days old. Lambs were distributed in a randomized block design. Five experimental diets were provided: Without supplementation, with supplementation but without the use of castor bean meal, and with gradual levels of replacement of soybean meal by detoxified castor bean meal (33%, 67%, and 100%). Higher values of DMI and nutrient digestibility (*p* < 0.05) were observed for animals that received supplements. The milk intake did not differ among the diets. The DMD showed a linear effect, while NDFD had a quadratic effect, depending on castor bean meal inclusion. The carcass yield did not differ between experimental diets. There was no significant effect on the urinary volume and metabolites investigated. In conclusion, the replacement of soybean meal by detoxified castor bean meal does not compromise animals’ development. Besides, the creep-feeding method positively affects lamb development, with higher body weight gain for supplemented animals.

## 1. Introduction

Vegetable oils are the most extensively used feedstocks for biodiesel production, of which castor bean oil stands out for its productive capacity [1,2]. The agro-chemistry industry uses two methods for oil extraction: Mechanical and chemical that generate solid residues. Mechanic methods form castor bean cake and chemical methods produce a castor bean meal [3].

Castor bean meal may cause feed intoxication due to the presence of the toxic component called ricin, which can causes abdominal pain, vomiting, severe dehydration, decreased urine production, and also promotes lower blood pressure in animals [4]. Specific damaging effects to the rumen and intestine have not been reported in the literature.

Castor bean meal, when detoxified, might be utilized in animal nutrition as a protein source, as it shows crude protein (CP) contents similar to soybean meal, which is the most frequent ingredient in dietary formulations for ruminants [5,6].

The commercial use of castor bean meal in animal feeding has been used for many decades. However, the studies were focused on maintenance and finishing, thus limiting the extrapolation of the conclusions for more specific categories, like wearing lambs. The maximization of performance during this phase results in an intensification of the productive system. Consequently, it allows the early weaning of the lambs at around 60 days of age. Thus, the concentrate intake could be applied from the 10th day of life. Besides, the ingestion of solids is essential to stimulate rumen development during this stage and needs to be conducted in controlled handlings, such as in a creep-feeding method [7].

Therefore, we hypothesized that castor bean meal, provided via a creep-feeding method, might fully replace soybean meal as the principal protein source in diets for weaning lambs, without interfering with the animals’ development.

Thus, this study aimed to evaluate the effect of replacing soybean meal by detoxified castor bean meal on intake and nutrient digestibility, body weight gain, carcass yield, physiological and urinary parameters, and the creep-feeding method effectiveness due to the inclusion of the castor bean meal.

## 2. Materials and Methods

The protocol n° 03/2011, entitled “Lambs supplementation via a creep-feeding method with diets containing detoxified castor bean meal”, was certified by the Ethics Committee for Animal Use of the Federal University of Viçosa (UFV), Viçosa, Brazil. The committee stated that the project followedthe ethical principles of animal experimentation, established by the Brazilian College of Animal Experimentation and with current legislation.

### 2.1. Castor Bean Meal Detoxification and Ricin Determination

The castor bean meal detoxification was performed according to the methodology proposed by Anandan et al. [8] using 40 g Ca(OH)2 kg^−1^ of castor bean meal and autoclaved at 15 psi for 60 min. The determination of the ricin content in detoxified castor bean meal was conducted according to Oliveira et al. [9] using a 0.5 M Tris-HCl buffer, pH 3.8.

The identification of the ricin fractions was made by gel electrophoresis, according to the method proposed by Laemmli [10]. Two stained methods were tested on the gels, a solution based on Coomassie Brilliant Blue G (1.5 g/L) and a silver nitrate solution (0.2 g/L). The identification of A (approximately 36 kDa) and B (approximately 29 kDa) ricin’s fractions was performed using molecular weight markers between 14 and 66 kDa (Sigma, Saint Louis, MO, USA). The detoxification method was considered valid only with the complete disappearance of the two ricin fractions [9].

### 2.2. Animals, Experimental Design and Diets

Forty-five male lambs, Dorper × Santa Inês, with an average initial body weight (IBW) of 7.95 kg, 15 days old at the beginning of the trial, were used. After birth, the umbilicus was cut and disinfected with a 10% iodine solution; immediately after, the ingestion of colostrum by the lamb was observed. Monitoring periodic parasitic diseases were achieved via fecal collection [11].

The adaptation to a solid diet started when the lambs were 10 days old and offered via a creep-feeding method. The trial period began when the lambs were 15 days old and ended when the lambs reached 75 days of age. The lambs were distributed in a randomized block design; the blocking factor was the date of entry into the experiment. Five experimental diets were applied, as follows:

Diet Pasture: Without concentrate supplementation;
Diet 0: Concentrate supplementation, without the addition of detoxified castor bean meal (DCBM);
Diet 33: Concentrate supplementation, replacing 33% of soybean meal by DCBM on a dry matter (DM) basis;
Diet 67: Concentrate supplementation, replacing 67% of soybean meal by DCBM on a DM basis;
Diet 100: Concentrate supplementation, replacing 100% of soybean meal by DCBM on a DM basis.

The animals were managed in semi-confinement; in this way, the lambs were gathered at night in five different groups (according to treatments) of nine lambs and separated from their mothers until the following morning. At this moment, the concentrate was provided ad libitum and weighed every day to allow 10% of refusals. The creep feeding was sized to allow simultaneous access to all lambs. In the morning, all lambs and mothers were conducted into a single paddock. The animals were rotated between two 3.5-ha paddocks during the experimental period according to forage availability. The mothers’ diet consisted of pastures, with a predominance of *Brachiaria decumbens* and a concentrate consisting of ground corn, soybean meal, and a mineral mixture with 14% crude protein. The lambs were weighed on the first and final days of the experimental period.

### 2.3. Laboratory Analysis

To determine the ingredients’ chemical composition (Table 1), supplements (Table 2), and refusals, samples of ingredients and refusals were collected weekly, conditioned in plastic bags, and frozen until laboratory analysis. Monthly, pasture samples were collected using the simulated grazing technique, in which the lambs grazing behavior was examined and afterwards was reproduced by the same trained researcher. The pasture samples were placed in plastic bags and frozen immediately for further analysis.

The samples were partially dehydrated in a forced ventilated oven at 55 °C for 72 h, after which they were ground in a knife mill using a 1-mm sieve. Chemical analysis was used to determine DM, mineral matter (Ash), CP, ether extract (EE), non-protein nitrogen (NPN), neutral detergent insoluble protein (NDIP), lignin, neutral detergent fiber corrected for ashes and protein (NDFap), and calcium according to Detmann et al. [12].

The total carbohydrates (TCs) were obtained according to Sniffen et al. [13]; the content of non-fiber carbohydrates (NFCs) corrected for ash and protein (NFCap) was calculated as proposed by Hall [14]; and the total digestible nutrients (TDNs) were obtained according to Weiss [15] (Table 1).

The milk was sampled and evaluated for fat, protein, lactose, and total solids (Table 3).

### 2.4. Intake and Apparent Digestibility of Nutrients

The estimation of the voluntary intake was made by using the indigestible neutral detergent fiber (iNDF) as an internal indicator, as proposed by Detmann et al. [16].

Milk intake was obtained according to the methodology proposed by Porto et al. [17]. The lambs were separated from their mothers for 12 h (18 to 6 h). During this period, the lambs were weighed and then contacted with their mothers for suckling. After 30 min of milk intake, the lambs were reweighed, allowing us to obtain milk intake by calculating the difference between the weights of the lambs before and after suckling and multiplying the difference by two. The total DM intake of the lambs was obtained by adding the pasture, the supplement, and the milk intake.

The digestibility trial started on the 7th day for 13 days, with an adaptation time of 7 days to the indicator (chromium oxide) and 6 days for fecal collection, at alternating hours (6, 8, 10, 12, 14, and 16 h). Two grams of the indicator were weighed and packed in paper cartridges and introduced in the esophagus via an applicator.

On the 7th digestibility trial day, pasture samples from the paddocks were collected by using the simulated grazing method [18]. The pasture samples were dried in a forced ventilation oven at 55°C for 72 h. Then, one part of each sample was ground in a Willey mill with a 1-mm sieve and the rest, using a 2-mm sieve.

The feces were collected directly from the rectum or immediately after excretion of the animals, avoiding contact with the ground or any other surface, taking approximately 10 g of fecal samples. The fecal dry matter (FDM) excretion was estimated according to Burns et al. [19], following the equation: FDM (g/day) = [quantity of the indicator provided (g)/concentration of the indicator at feces (%)] × 100.

The supplement intake was estimated using the following equation: SDMI (kg/day) = ((FDM × CMFe) / MSG) × SSG, where: CMFe = concentration of marker in the feces (kg/kg), MSG = marker present in the supplement supplied (kg/day), and SSG = supplement supplied (kg/day).

The dry matter intake (DMI) was estimated using the following equation: DMI (kg/day) = {[(FDM × CMF) - MS] / CMFo} + SDMI, where: CMF = concentration of marker in the feces (kg/kg), MS = intake of marker from supplement (kg), and CMFo = concentration of marker in the forage (kg/kg).

### 2.5. Slaughter and Carcass Yield

The animals were slaughtered at 75 days, after a 16-h period of solid and liquid fasting, and then the animals were weighed to obtain the slaughter body weight (SBW). Animals were stunned with the proper equipment, bled, skinned, and eviscerated. Immediately, the carcasses were weighed to obtain the hot carcass weight (HCW). The carcasses were conditioned in a cold chamber at 4°C for 24 h. After this period, the carcass was weighed again to obtain the cold carcass weight (CCW).

Blood, head, skin, hooves, tail, empty viscera (rumen-reticulum, omasum, abomasum, small intestine, and large intestine), mesentery, internal fat, organs (liver, heart, kidneys, spleen, lungs), as well as tongue, esophagus, trachea, and reproductive system weights were recorded and added to the HCW to obtain the empty body weight (EBW).

After obtaining the weights above, the true carcass yield (TCY), commercial carcass yield (CCY), biological carcass yield (BCY), and cooling losses (CL) were determined following equations from Cézar and Souza [20].

### 2.6. Urinary Excretion, Microbial Protein Synthesis, and Hepatic Enzymes

Spot urine was collected by spontaneous urination on the 42nd day of the experimental period. The collected urine was homogenized, filtered, and diluted, as described by Valadares et al. [21]. The samples were placed in plastic cups, labeled, and frozen for further analysis.

The total daily urinary volume was estimated, according to Tseu et al. [22]. The nitrogen balance was obtained by the difference between the total nitrogen ingested and the total nitrogen excreted in feces and urine. The total nitrogen (N) in feces and urine was determined according to the methodology described in Silva and Queiroz [23].

The microbial nitrogen (Nmic) production was estimated, according to Ushida et al. [24]. Analysis of purine derivatives (X-H, xanthine + hypoxanthine; A, allantoin; and UA, uric acid) was performed using a colorimetric method, according to Chen and Gomes [25].

The absorbed microbial purines (APs) were calculated from the excretion of purine derivatives (PDs) [26], and the intestinal flow of microbial nitrogen compounds was calculated according to the AP [25].

Blood samples were collected via the jugular vein using vacuum test tubes with gel separator. Immediately, they were centrifuged at 4000 rpm for 15 min. Then, serum samples were collected and preserved in glass containers and frozen at −15 °C. Subsequently, blood urea nitrogen and hepatic enzymes (AST, aspartate aminotransferase; ALT, alanine aminotransferase; and GGT, Gamma-glutamyl transferase) were determined using commercial kits.

### 2.7. Statistical Analysis

The trial was analyzed as a randomized complete block design using the date in which the animal’s entered into the experiment as a blocking factor, according to the following statistical model: Yijk = µ + Ti + Bj + eijk, where: Yij = experimental answer measured under the diet i, in the replication k of the j block, where k > 1; μ = general mean; Ti = effect of the experimental diets (previously described); Bj = effect of the block (date of entry into the experiment); eij = random error.

The sum of squares of the experimental diets was fractioned by orthogonal contrasts, following the scheme described in Table 4.

Data were analyzed using the statistic package SAS, Statistical Analysis System 9.3 [27], adopting 0.05 as the critical probability level for a type I error, and probability between 0.05 < *p* < 0.10 was considered as a tendency.

## 3. Results and Discussion

**Figure 1:**
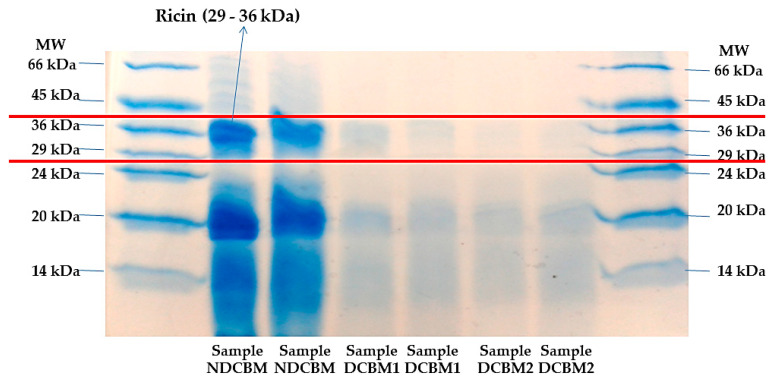
Gel stained with Coomassie brilliant blue. MW, Molecular weight; NDCBM, Non-DCBM; DCBM1, Sample 1 of DCBM; DCBM2, Sample 2 of DCBM.

**Figure 2:**
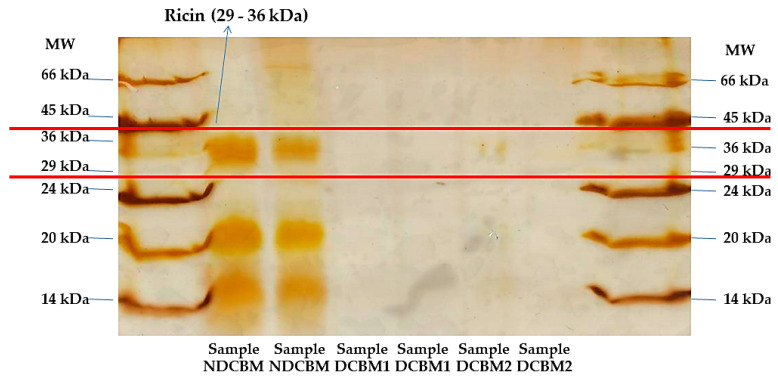
Gel stained with silver nitrate. MW, Molecular weight; NDCBM, Non-DCBM; DCBM1, Sample 1 of DCBM; DCBM2, Sample 2 of DCBM.

Coomassie brilliant blue coloration (CBC) (Figure 1) staining was more evident than silver nitrate (Figure 2) to stain the proteins in the gels. However, both had the same result, indicating that the treatment with calcium hydroxide was efficient in denaturing the ricin alkaloid.

The nutritional compound intakes are expressed in Table 5. Higher concentrate (DMIconc), DM (DMI), and CP (CPI) intakes (*p* < 0.05) were observed for animals that received supplementation with concentrate. Milk intake (DM basis) did not differ among the diets (*p* > 0.05). There was a linear (*p* = 0.001) and quadratic (*p* = 0.001) effect of the concentrate intake (DMIconc), showing that the inclusion of DCBM in the concentrate as a replacement for soybean meal, caused a reduction in the DMIconc. The pasture intake (DMI pasture) tended to a linear effect (*p* = 0.069). Although, the opposite effect was observed in comparison to the DMIconc. The inclusion of DCBM promoted an increase in the DMIpasture. The EE intake (EEI) and neutral detergent fiber intake (NDFI) did not differ statistically (*p* > 0.05) between treatments.

In the current study, performance differences occurred due to the intake of other feeds offered in the experiment. However, this increased DMIpasture is evidenced by the DMI. Therefore, only the diet without DCBM was lower than the others. The intake of the different nutritional compounds was lower than those reported by several authors [28,29,30]. We suggest that this difference was due to the animal category that was used, because the animals used in those experiments were heavier and older, and consequently had higher intakes. Silva [31], when working with lambs confined from the 14th day and that received mashed or pelleted feed via a creep-feeding method, found an average daily feed intake of 210 g for males.

The digestibility of the nutritional compounds is presented in Table 5. Averages did not differ between diets, except for a linear effect of the dry matter digestibility (DMD) and a quadratic effect tendency on the neutral detergent fiber digestibility (NDFD) (Table 5). So, the DMD and NDFD were higher than those reported in the literature [28,29,32] (Rodrigues et al., 2008). Possibly, the DMD is high due to the nursery, since the milk has excellent digestibility. The rumen development in this growth stage could explain the low NDFD. So, the fiber’s digestibility is compromised by the low fermentative capacity of the rumen [33,34]. The CPD and the EED were similar to those found by Sá et al. [35], but above those reported by Gionbelli et al. [29] and Rodrigues et al. [32]. In this latter case, only for the CPD, since the EED was not reported in the study.

The IBW (Table 6) did not differ between diets (*p* > 0.05), thereby indicating uniformity of the animals at the beginning of the experiment. The body weight gain (BWG) and average daily gain (ADG) showed a tendency (*p* = 0.053) of higher values for the use of the creep-feeding method and also presented quadratic and cubic effects. A cubic effect on feeding efficiency (FE) was observed (*p* = 0.017).

The IBW (Table 6) was uniform, which facilitates the visualization of the results, since all of the animals, on average, started the experiment with a similar body weight. BWG was reasonable, according to the range suggested by the NRC [36], from 50 to 100 g/day up to 350 to 400 g/day. The ADG was similar to that found by Poli et al. [37] for a weaned lamb. However, it was, on average, lower than values reported in the literature [32,35,38] and ranged from 112 to 277 g/day. However, this small difference between these values is possibly associated with the different diets and ages of the animals.

The quadratic effect can be understood due to a higher intake with the diets with 0% and 33% of DCBM inclusion. The impairment in the gain with the diet of 33% generated flexibility in the described parabola, whose mathematical function, in this case, resembles a cubic curve. The FE was significant for a cubic effect. The biological understanding of this contrast is similar to that observed for the variables BWG and ADG, considering that the FE is a function of gain and intake.

The TCY and CCY showed a cubic effect (*p* < 0.05). The BCY tended to increase with the increasing castor supplementation. A quadratic effect (*p* = 0.031) for CL was observed (Table 7).

The carcass yields (Table 7) were similar to those reported by Gionbelli et al. [29] and Ribeiro et al. [39]. When compared with other papers published in the literature, the carcass yields were similar to those observed by Vieira et al. [40], who worked with the replacement of soybean meal by detoxified castor bean meal at four levels (0%, 50%, 75%, and 100%), which are TCY (43.31%, 43.03%, 42.16%, and 45.10%), CCY (42.96%, 42.57%, 41.75%, and 44.08%), and BCY (56.10%, 55%, 05%, 53.63%, and 57.56%), respectively. The CL was higher in animals fed with diets without DCBM. Possibly, this is associated with the fat deposition profile in the carcass. Animals with higher subcutaneous fat thickness showed lower cooling losses [32]. The CL averages are in agreement with Gionbelli et al. [28].

The urinary volume (UV), xanthine-hypoxanthine (X-H), uric acid (UA) excretion, and purine derivative contents (PD), as well as the absorbed purines (APs) and urinary urea, did not show significant effects (*p* > 0.05) for any contrasts (Table 8). A tendency of a significant quadratic effect (*p* = 0.064) was observed in allantoin excretion.

Purine derivatives were lower than values reported in the literature and are possibly associated with protein deposition. So, it was reflected in the lower BWG of animals in this study. The protein might have been mobilized for other purposes, such as an immune response. The demand for amino acids increases when the animals have subclinical infections due to the need to repair damaged tissues and for a rapid immune response. The loss of the amino acid supply for other tissues significantly reduces anabolism, thereby compromising production rates [41,42].

The urine volume was similar to that found by Pereira et al. [43]. The levels of UA, X-H, and PD were lower than those reported by Pereira et al. [43]. The levels of A and PUR were similar to those suggested by these authors. The proportion of purines (A, UA, and X-H) relative to the total excreted PD was 49.1:1.2:49.1, respectively. This proportion was different from that reported by Oliveira et al. [9], as 85.2:10.4:4.4, and Pereira et al. [43], as 14.3:7.1:78.6. The urinary urea contents were well above those endorsed by Kaneko et al. [44] and ranged from 0 to 1.9 mg/dL for wearing lambs. However, the urinary excretion of urea was lower than that reported by Gionbelli et al. [29] and Pereira et al. [43] and similar to that suggested by Oliveira et al. [9].

The microbial nitrogen production (Nmic) and the N retained were not significant (*p* > 0.05) for any of the contrasts (Table 9). The use of concentrate supplementation increased the total N intake (*p* = 0.020) and N absorbed (*p* = 0.045). Higher inclusion levels of DCBM increased (*p* < 0.01) the efficiency of N use as a function of N intake (Effi1) and N absorbed (Effi2).

The flow of Nmic was lower than that found by Pereira et al. [43]. Possibly, this was due to the low ruminal fermentation capacity of the lambs used in this study. The percentages of N ingested, N absorbed, and N retained were lower than those reported in the literature [9,43,44]. We suggest that this is associated with the age of the lambs slaughtered in the weaning phase.

The serum levels of urea were significant (*p* = 0.039) for the quadratic effect. At the same time, there was a tendency of quadratic and linear effects for the enzymatic activities of alanine aminotransferase (ALT) and aspartate aminotransferase (AST) (*p* < 0.10), respectively. The Gamma-glutamyl transferase (GGT) level was not different (*p* > 0.10) for any of the contrasts (Table 10).

The liver condition was analyzed by determining the serum levels of AST, ALT, and GGT (Table 10), which are indicators of liver function, and their increase is related to liver damage. This ricin intoxication was evaluated in rats and sheep [45,46]. The values observed in the present experiment were similar to those reported by Rodrigues et al. [47], indicating that the liver function was not compromised.

The serum urea levels were above the levels found by Kaneko et al. [48], 8 to 20 mg/dL. The grain-based concentrate or forage with a high proportion of leaves are foods with highly soluble protein fractions. So, they were responsible for an increase in the ruminal ammonia production, which may be responsible for higher concentrations of serum urea [49].

The serum AST levels were in agreement with the level determined by Radostits et al. [50], from 60-280 IU/L, and by Kaneko et al. [48], from 0 to 90 IU/L. The AST levels were similar to those suggested by Gionbelli et al. [29] but higher than those reported by Rodrigues et al. [47]. Considering that the milk intake completed the nutritional requirements, some nutrients from solid feeds can be directed to gluconeogenesis, determining higher glucose levels, and a consequent higher activity of AST [49].

The enzymatic activities of ALT were low compared to those found by Radostits et al. [50], 22 to 28 IU/L, but normal values when compared to those found by Kaneko et al. [48], ranging from 0 to 30 IU/L. The enzymatic activities of ALT were lower than those valued found by Gionbelli et al. [29] and Oliveira et al. [9].

## 4. Conclusions

Castor bean meal has the potential for inclusion in diets for lambs because it does not affect the health, metabolism, and production performance of the animals. The replacement of soybean meal for DCBM does not affect the nutritional compound intake; however, higher levels of substitution increase the apparent digestibility of dry matter and efficient use of N with variable effects on BWG, ADG, and carcass yield.

Treatment of castor meal with 40 g of Ca (OH) 2/kg on a fresh matter basis in this experiment denatured ricin, corroborated by showing no detrimental effects on liver function in lambs. However, more research is required to elucidate further the effects of DCBM on the performance and health of wearing lambs.

The creep-feeding method positively affects lamb development, with higher BWG for supplemented animals.

## Figures and Tables

**Table 1:** Chemical composition of the ingredients used in the diets (% DM basis).

Item ^1^	Pasture 1 ^2^	Pasture 2 ^3^	Corn	Soybean Meal	DCBM ^4^	Urea + AS ^5^	Mineral Mixture ^6^
DM	20.60	30.06	88.39	89.60	89.77	97.60	99.99
Ash	7.29	7.78	1.26	5.40	9.42	-	-
CP	18.53	11.09	9.40	51.00	35.78	265.78	-
EE	2.36	1.45	0.53	2.22	0.38	-	-
NDFap	41.56	52.87	35.63	28.60	35.61	-	-
iNDF	12.62	21.18	2.32	1.73	42.61	-	-
Lignin	-	8.05	-	-	-	-	-
NDIP	18.69	12.88	10.71	34.14	22.36	-	-
NFCap	30.26	26.81	53.18	12.79	18.81	-	-
NPN	-	6.32	6.25	34.56	24.23	-	-
Ca	0.33	0.49	0.03	0.39	2.03	-	-

^1^ DM, Dry matter (% Fresh matter basis); CP, Crude protein; EE, Ether extract; NDFap, Neutral detergent fiber corrected for ash and protein; iNDF, Indigestible neutral detergent fiber; NDIP, Neutral detergent-insoluble protein; NFCap, Non-fiber carbohydrates corrected for ash and protein; NPN, Non-protein nitrogen; TDN, Total digestible nutrients. ^2,3^ *Brachiaria decumbens* pasture of paddock 1 and 2; ^4^ DCBM, Detoxified castor bean meal; ^5^ Urea + AS, Urea + ammonium sulfate in proportion of 9:1, with urea containing 47.25% of nitrogen; ^6^ Macromineral composition (%): Ca = 24.61, P = 10.87, Mg = 1.28, Na = 4.50, K = 0.25 and S = 2.41. Micromineral composition (mg/kg of DM): Co = 21.07, Cu = 576.8, Fe = 1045.4, Mn = 362.7 and Zn = 2569.8.

**Table 2:** Proportion of ingredients and chemical composition of the supplements.

Item ^1^	Detoxified Castor Bean Meal (%)			
	0	33	67	100
Proportion (% DM basis)				
Ground corn	65.84	65.84	66.03	66.03
Soybean meal	32.02	21.41	10.60	-
Detoxified castor bean meal	-	10.01	20.01	30.01
Urea	-	0.55	1.09	1.64
Ammonium sulfate	-	0.06	0.12	0.18
Mineral mixture	2.14	2.14	2.14	2.14
Chemical composition (% DM basis)				
DM	88.97	89.03	89.09	89.16
Ash	2.56	2.93	3.29	3.66
CP	22.52	22.30	22.00	21.79
EE	1.06	0.86	0.66	0.47
NDFap	32.62	33.15	33.69	34.21
iNDF	2.08	6.16	10.24	14.32
NDIP	17.98	16.60	15.17	13.78
NFCap	41.25	41.17	41.17	41.10
TC	73.86	73.91	74.05	74.09
NPN	15.18	13.94	12.64	11.40
Ca	0.14	0.31	0.47	0.63
TDN	91.76	91.55	91.35	91.14

^1^ DM, Dry matter (% Fresh matter basis); CP, Crude protein; EE, Ether extract; NDFap, Neutral detergent fiber corrected for ash and protein; iNDF, Indigestible neutral detergent fiber; NDIP, Neutral detergent-insoluble protein; NFCap, Non-fiber carbohydrates corrected for ash and protein; TC, Total carbohydrates; NPN, Non-protein nitrogen; TDN, Total digestible nutrients.

**Table 3:** Composition of milk (%) consumed by lambs supplemented with or without castor bean meal via a creep-feeding method.

Item	Pasture	Detoxified Castor Bean Meal (%)			
		0	33	67	100
Fat	5.12	5.31	7.17	5.61	5.15
Protein	5.51	5.91	5.85	5.61	5.81
Lactose	4.98	4.55	4.81	4.56	4.69
Total solids	16.12	16.45	18.42	16.10	16.34

**Table 4:** Distribution of the coefficients used in the contrasts.

Contrasts ^1^	Pasture	Detoxified Castor Bean Meal (%)			
		0	33	67	100
CO	+4	−1	−1	−1	−1
L	-	−3	−1	+1	+3
Q	-	+1	−1	−1	+1
C	-	−1	+3	−3	+1

^1^ CO, Pasture vs. supplementation; L, Linear, Q, Quadratic, and C, Cubic effects, refers to the replacement of soybean meal with detoxified castor bean meal.

**Table 5:** Nutrient intake and apparent digestibility (mean ± SD) of diets supplemented with or without castor bean meal via a creep-feeding method.

Item ^1^	Pasture	Detoxified Castor Bean Meal (%)				*p*-Value ^2^			
		0	33	67	100	CO	L	Q	C
Intake (g d^−1^)									
Milk intake	24.4 ± 9.5	33.8 ± 9.4	30.5 ± 9.8	35.8 ± 9.3	39.7 ± 1.0	0.214	0.509	0.643	0.777
DMIconc	0.0	95.9	58.9	34.1	28.6	0.001	0.001	0.001	0.296
DMIpasture	249 ± 19.5	230 ± 13.0	238 ± 12.2	256 ± 11.5	280 ± 12.2	0.933	0.069	0.706	0.977
DMI	276 ± 22.7	321 ± 22.7	330 ± 24.0	326 ± 22.7	352 ± 24.0	0.033	0.403	0.706	0.684
CPI	38.4 ± 3.5	51.9 ± 3.5	50.3 ± 3.7	47.3 ± 3.5	50.3 ± 3.7	0.005	0.620	0.526	0.656
EEI	6.3 ± 0.6	6.6 ± 0.6	7.5 ± 0.6	7.0 ± 0.6	7.7 ± 0.6	0.157	0.260	0.853	0.352
NDFI	132 ± 10.1	121 ± 9.5	132 ± 10.1	132 ± 9.5	141 ± 10.1	0.971	0.165	0.955	0.651
Apparent digestibility (%)									
DMD	90.4 ± 1.7	85.9 ± 1.7	88.5 ± 1.8	90.8 ± 1.7	90.4 ± 1.8	0.441	0.045	0.397	0.754
CPD	84.4 ± 3.4	86.0 ± 3.4	89.8 ± 3.5	88.0 ± 3.4	89.4 ± 3.5	0.126	0.407	0.598	0.391
EED	80.4 ± 3.1	80.5 ± 3.4	82.7 ± 3.2	81.5 ± 3.1	84.0 ± 3.2	0.500	0.394	0.953	0.517
NDFD	11.8 ± 0.6	12.6 ± 0.6	12.0 ± 0.6	11.5 ± 0.5	12.7 ± 0.6	0.402	0.894	0.062	0.426

^1^ Milk intake (Dry matter basis); DMIconc, DMI from concentrate; and DMIpasture, DMI from pasture ^2^ Contrasts; CO, Pasture vs. supplementation; L, Linear, Q, Quadratic and C, Cubic effects, refers to the replacement of soybean meal with detoxified castor bean meal.

**Table 6:** Performance of lambs (mean ± SD) supplemented with or without castor bean meal via a creep-feeding method.

Item ^1^	Pasture	Detoxified Castor Bean Meal (%)				*p*-Value ^2^			
		0	33	67	100	CO	L	Q	C
IBW (kg)	7.8 ± 1.6	7.9 ± 1.6	8.0 ± 1.6	7.9 ± 1.6	8.2 ± 1.6	0.817	0.811	0.850	0.878
BWG (kg)	4.8 ± 1.9	7.6 ± 1.9	3.8 ± 1.9	7.7 ± 1.9	6.4 ± 1.9	0.053	0.941	0.087	0.0002
ADG (g d−1)	80.8 ± 31.6	126.1 ± 31.5	63.8 ± 31.8	127.8 ± 31.5	106.0 ± 32.2	0.053	0.941	0.087	0.0002
FE (%) ^3^	40.5 ± 15.0	52.6 ± 14.9	20.9 ± 15.2	43.8 ± 14.9	32.1 ± 15.4	0.714	0.280	0.213	0.017

^1^ IBW, Initial body weight; BWG, Body weight gain; ADG, Average daily gain; FE, Feed efficiency. ^2^ Contrasts; CO, Pasture vs. supplementation; L, Linear; Q, Quadratic; and C, Cubic effects; refers to the replacement of soybean meal with detoxified castor bean meal. ^3^ FE = (ADG/DMI) × 100.

**Table 7:** Carcass yield (%) of lambs (mean ± SD) supplemented with or without castor bean meal via a creep-feeding method.

Item ^1^	Pasture	Detoxified Castor Bean Meal (%)				*p*-Value ^2^			
		0	33	67	100	CO	L	Q	C
TCY	43.6 ± 2.8	44.2 ± 2.8	41.3 ± 2.8	45.8 ± 2.8	44.7 ± 2.9	0.771	0.333	0.497	0.034
CCY	42.1 ± 3.2	42.2 ± 3.2	40.2 ± 3.3	44.8 ± 3.2	43.1 ± 3.3	0.777	0.239	0.925	0.042
BCY	65.1 ± 1.9	62.5 ± 1.9	61.9 ± 2.0	65.3 ± 1.9	64.5 ± 2.0	0.229	0.077	0.962	0.121
CL	3.5 ± 1.2	5.1 ± 1.2	2.5 ± 1.2	2.4 ± 1.2	3.3 ± 1.2	0.822	0.115	0.031	0.664

^1^ TCY, True carcass yield; CCY, Commercial carcass yield; BCY, Biological carcass yield; CL, Cooling loss. ^2^ Contrasts; CO, Pasture vs. supplementation; L, Linear; Q, Quadratic; and C, Cubic effects; refers to the replacement of soybean meal with detoxified castor bean meal.

**Table 8:** Urine parameters of lambs (mean ± SD) supplemented with or without castor bean meal via a creep-feeding method.

Item ^1^	Pasture	Detoxified Castor Bean Meal (%)				*p*-Value ^2^			
		0	33	67	100	CO	L	Q	C
UV (L d^−1^)	1.5 ± 0.3	1.4 ± 0.3	1.4 ± 0.3	1.5 ± 0.3	1.5 ± 0.3	0.921	0.615	0.962	0.772
X-H (mmol d^−1^)	9.1 ± 1.5	9.7 ± 1.5	9.0 ± 1.6	8.9 ± 1.5	9.1 ± 1.6	0.924	0.759	0.735	0.974
A (mmol d^−1^)	8.4 ± 2.6	12.9 ± 2.5	7.8 ± 2.7	6.4 ± 2.5	9.4 ± 2.8	0.755	0.226	0.064	0.952
UA (mmol d^−1^)	0.2 ± 0.1	0.2 ± 0.1	0.2 ± 0.1	0.2 ± 0.1	0.2 ± 0.1	0.614	0.755	0.161	0.235
PD (mmol d^−1^)	18.0 ± 3.3	22.9 ± 3.3	17.2 ± 3.5	15.4 ± 3.3	19.4 ± 3.6	0.846	0.407	0.150	0.893
AP (mmol d^−1^)	18.3 ± 3.7	23.7 ± 3.7	17.5 ± 4.0	14.8 ± 3.7	19.9 ± 4.0	0.882	0.419	0.153	0.805
Urinary urea (g d^−1^)	10.0 ± 2.6	11.4 ± 2.6	10.7 ± 2.7	10.2 ± 2.6	6.8 ± 2.7	0.908	0.102	0.484	0.719

^1^ UV, Urinary volume; A, Allantoin; X-H, Xanthine-hypoxanthine; PD, Purine derivatives content; AP, Absorbed microbial purines. ^2^ Contrasts; CO, Pasture vs. supplementation; L, Linear; Q, Quadratic; and C, Cubic effects; refers to the replacement of soybean meal with detoxified castor bean meal.

**Table 9:** Nitrogen balance of lambs (mean ± SD) supplemented with or without castor bean meal via a creep-feeding method.

Item ^1^	Pasture	Detoxified Castor Bean Meal (%)				*p*-Value ^2^			
		0	33	67	100	CO	L	Q	C
Nmic (gN d^−1^)	13.3 ± 2.7	17.2 ± 2.7	12.7 ± 2.9	10.8 ± 2.7	14.5 ± 2.9	0.882	0.419	0.153	0.805
N intake (g d^−1^)	6.7 ± 0.5	8.3 ± 0.5	8.0 ± 0.5	7.6 ± 0.5	8.0 ± 3.3	0.020	0.560	0.456	0.601
N absorbed (g d^−1^)	6.0 ± 0.5	7.2 ± 0.5	7.2 ± 0.5	6.7 ± 0.5	7.2 ± 0.5	0.045	0.759	0.639	0.513
N retained (g d^−1^)	3.1 ± 0.5	3.8 ± 0.5	3.3 ± 0.6	3.9 ± 0.5	4.8 ± 0.6	0.173	0.157	0.193	0.693
Effi1 (%Nint)	46.0 ± 5.6	40.4 ± 5.6	40.5 ± 5.9	50.3 ± 5.6	59.8 ± 5.9	0.750	0.008	0.385	0.606
Effi2 (%Nabs)	51.4 ± 5.8	45.5 ± 5.8	44.5 ± 6.2	56.8 ± 5.8	66.8 ± 6.2	0.774	0.009	0.402	0.686

^1^ Nmic, Microbial nitrogen; Effi1, N use as a function of N intake; and Effi2, N use as a function of N absorbed. ^2^ Contrasts; CO, Pasture vs. supplementation; L, Linear; Q, Quadratic; and C, Cubic effects; refers to the replacement of soybean meal with detoxified castor bean meal.

**Table 10:** Metabolic status of lambs (mean ± SD) supplemented with or without castor bean meal via a creep-feeding method.

Item ^1^	Pasture	Detoxified Castor Bean Meal (%)				*p*-Value ^2^			
		0	33	67	100	CO	L	Q	C
Serum urea (mg dL^−1^)	32.4 ± 5.9	34.2 ± 5.9	56.4 ± 7.7	37.6 ± 5.9	33.6 ± 6.3	0.209	0.496	0.039	0.060
ALT (IU L^−1^)	8.6 ± 1.6	9.6 ± 1.6	13.4 ± 2.1	10.2 ± 1.6	8.1 ± 1.7	0.329	0.315	0.096	0.328
AST (IU L^−1^)	97.0 ± 9.1	105 ± 9.1	92.0 ± 12.2	84.0 ± 9.1	81.9 ± 9.7	0.559	0.070	0.573	0.996
GGT (IU L^−1^)	61.2 ± 13.4	55.4 ± 13.3	78.8 ± 17.2	53.4 ± 13.2	45.3 ± 14.5	0.832	0.339	0.258	0.311

^1^ AST, Aspartate-aminotransferase; ALT, Alanine-aminotransferase; GGT, Gamma-glutamyltransferase. ^2^ Contrasts; CO, Pasture vs. supplementation; L, Linear; Q, Quadratic; and C, Cubic effects; replacement of soybean meal by detoxified castor bean meal.
